# The first preparative solution phase synthesis of melanotan II

**DOI:** 10.3762/bjoc.4.39

**Published:** 2008-10-30

**Authors:** Vladimir V Ryakhovsky, Georgy A Khachiyan, Nina F Kosovova, Elena F Isamiddinova, Andrey S Ivanov

**Affiliations:** 1 R&D Dept., Pharm-Sintez, 38 Shosse Entusiastov, 111123 Moscow, Russia

**Keywords:** melanotan II, solution phase synthesis, peptide synthesis

## Abstract

Melanotan II is a synthetic cyclic heptapeptide used to prevent a sunlight-induced skin cancer by stimulating the skin tanning process. In this paper we report the first solution phase synthesis of the title compound. The hexapeptide sequence has been assembled by [(2+2)+1+1] scheme. After removing the orthogonal protection, a carbodiimide mediated lactamization, involving the ε-amino group of lysine and γ-carboxy group of aspartic acid, led to a cyclic intermediate. Appending *N*-acetylnorleucine concluded the assembly of melanotan II molecule. Protection of the lateral groups in arginine and tryptophan was omitted for atom and step economy reasons. The total synthesis of melanotan II was accomplished in 12 steps with 2.6% overall yield, affording >90% pure peptide without using preparative chromatography.

## Introduction

Development of solid phase peptide synthesis methodology [1], recombinant techniques for expressing peptides and proteins in microorganisms [2], and most recently methods for producing peptides and proteins in transgenic animals [3] and plants [4], have greatly increased the availability of peptide compounds. However, the classical solution phase approach still retains its usefulness, especially when performed on a large scale. Novel powerful solvent systems combined with special protection tactics allow for even proteins to be synthesized in solution. The 136-residue human pleiotrophin and the 238-residue *Aequoria* green fluorescent protein are examples of such syntheses [5]. Most of the approved peptide pharmaceuticals are currently produced by chemical synthesis in solution including oxytocin, adrenocorticotropic hormone (ACTH), desmopressin, leuprolide, goserelin, and octreotide [6]. There is no account of a solution phase synthesis of melanotan II. The present paper describes a classical approach to this important therapeutical heptapeptide in full detail. Special attention is paid to minimum orthogonal protection of lateral functional groups to achieve maximum atom [7–8] and step [9] economy.

**Figure 1:**
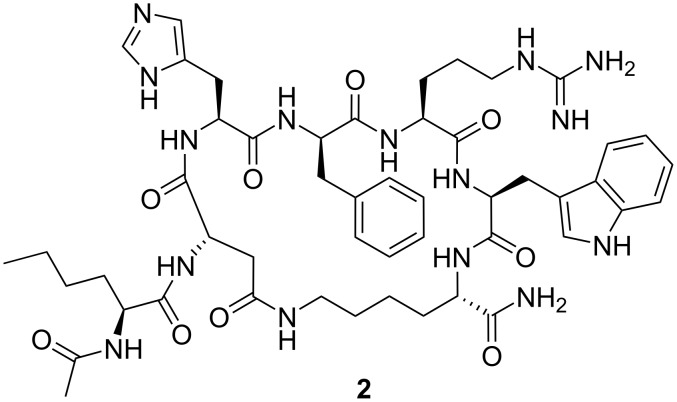
The structure of melanotan II.

α-Melanocyte stimulating hormone (α-MSH, α-melanotropin) is a linear tridecapeptide of the formula Ac-Ser-Tyr-Ser-Met-Glu-His-Phe-Arg-Trp-Gly-Lys-Pro-Val-NH*_2_* (**1**), involved in the regulation of skin pigmentation. Production of this hormone is stimulated by irradiation of skin by the sun’s ultraviolet rays. The α-MSH triggers the skin tanning, a process in which skin tanning cells (melanocytes) produce skin tanning pigment (melatonin). Since tan is a body’s natural protection against the ultraviolet, stimulation of melanogenesis process with exogenic hormone prior to irradiation would be a good protection against the UV-induced skin cancer. Unfortunately, the native hormone, α-MSH, was found to be too unstable *in vivo* to be used as a therapeutical agent. Once the tetrapeptide Ac-His-Phe-Arg-Trp-NH_2_ was identified as a “message sequence” of melanotropin, responsible for minimal physiological action in the frog [10] and lizard [11], a wide range of analogs was synthesized by the group of V. Hruby at University of Arizona [12–19] and other research groups [20–23]. Modification of the α-MSH structure, including replacement of the oxidizable L-methionine with isosteric L-norleucine, replacement of L-phenylalanine with its enantiomer D-phenylalanine, and locking of the linear peptide sequence in its biologically active conformation by lactamization of the lysine ε-amino group and glutamic acid γ-carboxy group, led to a cyclic pseudopeptide analog of α-MSH with good metabolic stability and exceptional activity, known as melanotan II (**2**) (Figure 1).

## Results and Discussion

To date, several accounts of solid phase synthesis of peptide **2** have been reported. A representative one, reported by Hruby and co-workers, exploited *p*-methylbenzhydrylamine resin (pMBHA) as the solid support and *tert*-butyloxycarbonyl (Boc) tactics for temporary protection of α-amino groups. The ε-amino group of lysine and the γ-carboxy group of aspartic acid, involved in lactamization, were protected as the base-cleavable Fmoc amide and Fm ester respectively. After synthesizing the peptide chain and cleavage of the base-labile protecting groups, an efficient on-resin cyclization was performed using an excess of BOP as the coupling agent. Attaching of *N*-Boc-norleucine, removal of Boc, acetylation of norleucine amino group with acetic anhydride, HF mediated cleavage of the resulting peptide from the polymer support, and finally purification using RP-HPLC afforded the target compound **2** in 55–60% overall yield [18,24]. The yield dropped to 30% when lactamization of the linear heptapeptide sequence, synthesized on the resin, was performed in DMF solution using DPPA/K_2_HPO_4_.

Despite the high overall yield in the described solid phase approach, it has several drawbacks for the scale-up process such as the application of the highly toxic and corrosive hydrogen fluoride for cleavage of the peptide from the resin, low loading (0.30–0.35 mmol/g of resin) proved necessary for successful on-resin cyclization, and the use of excess amounts of reagents (3-fold of DIC, 2.4-fold of HOBt, etc.) on each step.

**Scheme 1:**
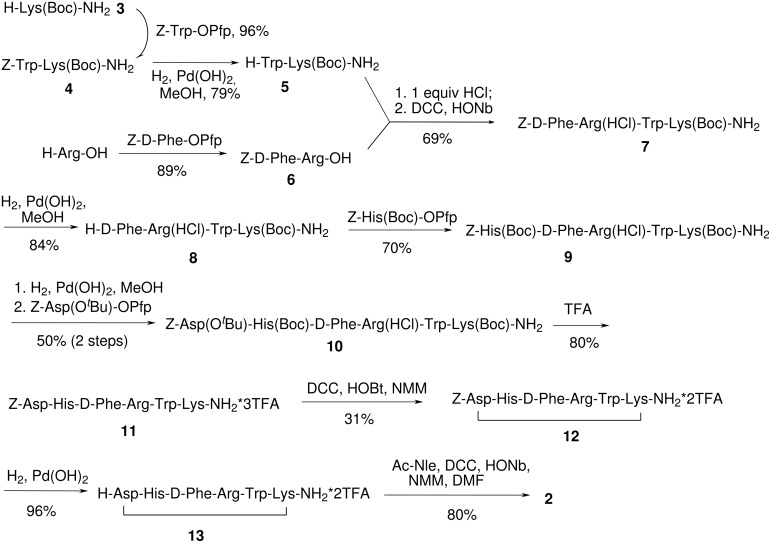
Synthesis of melanotan II.

Our plan for the solution phase synthesis of peptide **2** was based on the [(2+2)+1+1] scheme for assembly of the linear hexapeptide backbone using benzyloxycarbonyl (Z) group as the temporary protection for amino acids’ *N*-termini (Scheme 1). The presence of aspartic acid in the target molecule poses a potential problem, due to its susceptibility to base-catalyzed cyclization to aspartimide. Esterification of γ-carboxy group does not fully protect aspartic acid from this unwanted process [25]. Aspartimides are known to readily racemize under basic conditions [26], and undergo ring-opening reactions with nucleophiles, leading to formation of a variety of by-products. Thus, attack of nucleophiles yields predominately β-aspartyl peptide derivatives. This deleterious side process usually takes place when the synthesis is based on Fmoc tactics, wherein large excess secondary amines are employed for deprotection [27–29]. Bearing this in mind, we postponed appending aspartic acid to a late stage of the synthesis, used only equimolar quantities of *N*-methylmorpholine (NMM) when a base was required for a reaction, and avoided any base-cleavable protecting groups, such as Fmoc or formyl. Despite concerns that the indole nitrogen would be susceptible to attack by *tert*-butyl cation generated upon Boc-group cleavage, we found that protection of indole nitrogen in tryptophan could be omitted from this process without substantial deterioration of the product yield or purity. That allowed us to decrease the overall length of the synthesis by two steps. The ε-amino group of lysine and the γ-carboxy group of aspartic acid were protected as Boc amide and *tert*-butyl ester respectively. Thus, all the protecting groups we used were cleavable either under acidic or hydrogenolytic conditions, releasing only volatile by-products, and all the reagents used were relatively inexpensive. These two points are very advantageous for the preparative synthesis. Another feature of our synthetic plan was to keep the side-chain functionality of arginine unprotected. In order to suppress the nucleophilic nature of the guanidine group in arginine, it was deactivated as the monohydrochloride salt over the course of 4 steps, and then as trifluoroacetate for another 2 steps. A similar method for arginine deactivation had been applied earlier in the first solution-phase synthesis of ACTH [30].

The assembly of the melanotan II molecule was started by coupling of *N*^ε^-Boc-lysinamide (**3**) with *N*^α^-benzyloxycarbonyltryptophan pentafluorophenyl ester to yield dipeptide **4** (Scheme 1). Cleavage of the Z protecting group afforded dipeptide **5**. Reaction of arginine with *N*^α^ -Z-D-phenylalanine pentafluorophenyl ester led to a protected dipeptide **6**. Since arginine exists in DMF solution in zwitterionic form, no protection for highly basic guanidine group was required. The guanidine group of arginine was deactivated for the next 4 steps by adding an equivalent of HCl (dioxane solution) to dipeptide **6**. The resulting salt was coupled with another dipeptide **5** using a combination of *N*,*N*′-dicyclohexylcarbodiimide (DCC) and *N*-hydroxynorbornene-2,3-dicarboximide (HONb) to yield a tetrapeptide product **7**. The latter was subjected to catalytic hydrogenolysis leading to tetrapeptide salt **8** with an uncapped N-terminus. Hydrochloride of tetrapeptide **8** was coupled with His(*N*^α^-Boc,*N*^T^-Z)-OPfp in methyl alcohol to produce pentapeptide **9**. Further chain elongation was effected by de-blocking the N-terminus in pentapeptide **9** and coupling of the resulting product with *t*-butyl *N*^α^-benzyloxycarbonylaspartate to give hexapeptide **10**. One-step cleavage of all acid-labile groups with excess of trifluoroacetic acid (TFA) yielded the tris-trifluoroacetate salt of deprotected linear hexapeptide **11** with all prerequisites for cyclization. The cyclization step, involving the ε-amino group of lysine and γ-carboxy group of aspartic acid, was performed using an 8-fold excess of DCC as the coupling agent and 1-hydroxybenzotriazole (HOBt) as a racemization suppressant. The yield of the cyclized product **12** was 31%, very close to the reported 30% yield for solution-phase cyclization of a related linear heptapeptide obtained by solid phase peptide synthesis [18]. The total synthesis of melanotan II was concluded by coupling *N*-acetylnorleucine to the cyclic hexapeptide using a combination of DCC and HONb (Scheme 1). The target compound **2** obtained was >90% pure by HPLC (UV). LC-MS analysis confirmed the identity of molecular masses and retention times for the synthesized product and melanotan II purchased from a commercial source. Electrospray injection mass spectrometry of both samples demonstrated two characteristic peaks with *m/z* 512 and 1024, corresponding to [M+2H]^2+^ and [M+H]^+^ respectively.

## Conclusion

In conclusion, we have developed the first solution phase synthesis of the cyclic heptapeptide melanotan II in 2.6% overall yield for 12 steps. Full-scale optimization of the process is being investigated and the results will be reported in due course.

## Experimental

A melanotan II sample was purchased from Fluorochem Ltd. (Cat. code M02830). All reagents and solvents were obtained from commercial sources except for the protected pentafluorophenyl esters: ^α^Z-Trp-OPfp, Z-D-Phe-OPfp, Z-His(^T^Boc)-OPfp, ^α^Z-Lys(^ε^Boc)-NH2, and Z-Asp(O*^t^*Bu)OPfp, which were prepared in-house according to the well established procedures [31–32]. Diethyl ether and tetrahydrofuran were distilled from sodium wire prior to use, and showed negative peroxide test with potassium iodide. ^1^H NMR spectra were recorded with a Bruker AMX-360 spectrometer. LC-MS analysis was performed on Agilent 1200 instrument with Agilent 6310 ion trap LC-MS detector.

### Z-Trp-Lys(^ε^Boc)-NH_2_ (**4**)

Z-Trp-OPfp (160 g, 317.2 mmol) was added to a stirred solution of *N*^ε^-Boc-lysinamide (61.4 g, 250.3 mmol) in 1.5 L of THF/MeOH (4 : 1 v/v). After an hour of stirring at r.t., the solvent was removed under vacuum, the residue stirred in hexane-ether (9 : 1 v/v), and the precipitate filtered off. The crude product, containing some pentafluorophenol and Z-Trp-OPfp, was re-suspended in 2 L of ethyl acetate/hexane (1 : 1 v/v), stirred for 1 h, filtered, and air-dried to provide the 95% pure product (147.5 g, 260.7 mmol). Yield 96%. ESI-MS: *m/z* 566.5 [M+H]^+^. ^1^H NMR (DMSO-d_6_): δ 10.78 (s, 1H, NH_indole_); 7.87 (d, 1H, *J* = 8.28); 7.63 (d, 1H, *J* = 8.28); 6.9–7.4 (m, 11H, 9CH_Ph,indole_, 2NH); 6.68 (br s, 1H); 4.95 (s, 2H, CH_2_Ph); 4.30 (m, 1H, αCH Trp); 4.17 (m, 1H, αCH Lys); 3.2–2.70 (m, 4H, βCH_2_ Trp, εCH_2_ Lys); 1.37 (s, 9H, *^t^*Bu); 1.0–1.65 (m, 6H, β,γ,δCH_2_ Lys).

### H-Trp-Lys(^ε^Boc)-NH_2_ (**5**)

Pd(OH)_2_ (4 g) was added to a solution of **4** (147 g, 260.0 mmol) in methanol (2 L), and the mixture was stirred at r.t. overnight under the hydrogen atmosphere. The solvent was removed under reduced pressure, and the residue was stirred with diethyl ether, filtered, and air-dried to give 89 g of the product. Yield 79%. ESI-MS: *m/z* 432.3 [M+H]^+^. ^1^H NMR (DMSO-d_6_): δ 10.83 (s, 1H, NH_indole_); 7.96 (d, 1H, *J* = 8.28 Hz); 7.56 (d, 1H, *J* = 7.92 Hz); 7.34 (s, 1H); 7.33 (d, 1H, *J* = 8.28); 7.17 (s, 1H); 7.06 (t, 1H, *J* = 7.92 Hz); 6.99 (s, 1H); 6.96 (t, 1H, *J* = 7.92 Hz, CH_arom_); 6.71 (br s, 1H, NH); 4.20 (m, 1H, αCH); 3.48 (m, 1H, αCH); 3.15–2.7 (m, 4H, βCH_2_ Trp, εCH_2_ Lys); 1.37 (s, 9H, *^t^*Bu); 1.0–1.8 (m, 6H, β,γ,δCH_2_ Lys).

### Z-D-Phe-Arg-OH (**6**)

L-arginine (87.3 g, 501 mmol) and Z-D-Phe-OPfp (240 g, 516 mmol) were dissolved in DMF (1.5 L) and the solution was stirred for 20 h at r.t. The solvent was removed under vacuum, and the residue partitioned in 1.3 L of chloroform/water (3.3 : 1 v/v). The chloroform layer was separated, washed with water (0.3 L), dried over magnesium sulphate, and evaporated to dryness. The residue was stirred with ether, filtered and air-dried to give 220 g of dipeptide **6**. Yield 89%. ESI-MS: *m/z* 456.3 [M+H]^+^. ^1^H NMR (DMSO-d_6_): δ 8.82 (br s, 1H, NH); 7.50–7.75 (m, 5H, 5NH); 7.10–7.35 (m, 10H, 2Ph); 4.94 (dd, 2H, OCH_2_Ph); 4.27 (m, 1H, αCH D-Phe); 3.97 (m, 1H, αCH Arg); 2.73 (t, 1H, CH_2_Ph Phe); 1.55–1.7 (m, 2 H, CH_2_ Arg); 1.38 (t, 2H, CH_2_ Arg).

### Z-D-Phe-Arg(HCl)-Trp-Lys(^ε^Boc)-NH*_2_* (**7**)

A solution of HCl in dioxane (17%, 50.3 mL as calculated from the acid-base titration of product **6** obtained from the previous step) was added dropwise to a stirred solution of protected dipeptide **6** (134.5 g, 295.3 mmol) in DMF (800 mL) at 7 °C. Then HONb (79.4 g, 179.2 mmol) and dipeptide **5** (115.8 g, 268.5 mmol) were added, and the mixture was stirred for several minutes. A solution of DCC (61 g, 295 mmol) in DMF (220 mL) was added dropwise while stirring, and the mixture was stirred overnight at r.t. The precipitate of *N*,*N*′-dicyclohexylurea (DCU) was filtered off, washed with cold DMF, and the combined DMF solutions were evaporated to dryness under vacuum. The residue was partitioned in of water/ethyl acetate/THF system at 40 °C (0.6 : 0.6 : 0.7 L). The organic layer was separated and washed with water (0.6 L). The combined aqueous solutions were extracted with THF/ethyl acetate (0.5 : 0.5 L), and the combined organic layers were evaporated to dryness. The residue was stirred with ether (1 L), filtered, and dried to provide 185 g of the product. Yield 69%.

### H-D-Phe-Arg(HCl)-Trp-Lys(^ε^Boc)-NH_2_ (**8**)

Protected tetrapeptide **7** (239 g, 263.9 mmol) was dissolved in methanol (1.4 L), and Pd(OH)_2_ (6.6 g) was added. The mixture was stirred at r.t. under hydrogen atmosphere. Pd(OH)_2_ was removed by filtration, and the filtrate was evaporated under vacuum, the residue was dissolved in THF at 40 °C, the solution was cooled to r.t., and the product was precipitated by addition of ethyl acetate (1.6 L). The precipitate was filtered, re-suspended in ether, filtered, and air-dried to give 170.5 g of the crude material. Yield 84%. ESI-MS: *m/z* 735.6 [M+H]^+^.

### Z-His(Boc)-D-Phe-Arg(HCl)-Trp-Lys(^ε^Boc)-NH_2_ (**9**)

A solution of tetrapeptide hydrochloride **8** (34 g, 44.1 mmol) in methanol was added to Z-His(^T^Boc)-OPfp (22.2 g, 48.8 mmol). After 5 min of stirring the resulting solution was left in the fridge overnight. Methanol was removed under reduced pressure, the residue triturated with ether, filtered, dissolved in chloroform at 40 °C (900 mL), and washed with water (2 × 900 mL). The organic layer was separated, dried over magnesium sulphate, and evaporated to dryness under vacuum. The residue was stirred with ether, filtered, and air-dried to provide 32 g of the crude product. Yield 70%. Purity 85% by HPLC.

### Z-Asp(O^t^Bu)-His(Boc)-D-Phe-Arg(HCl)-Trp-Lys(^ε^Boc)-NH_2_ (**10**)

The crude product **9** (32 g, 30.69 mmol), obtained from the previous step, was dissolved in methanol (600 mL), then Pd(OH)_2_ (1 g) was added and the mixture was hydrogenated with hydrogen while stirred at r.t. for 24 h. To the reaction mixture, containing deprotected pentapeptide hydrochloride and Pd(OH)_2_, was added a solution of Z-Asp(^γ^O*^t^*Bu)-OPfp (26.3 g) in THF (150 mL), and the resulting mixture was stirred for 1 h at r.t. The solvent was evaporated under vacuum, the residue stirred with ether, filtered, washed with ether, and dried. The raw material was partitioned in THF/ethyl acetate/water (1.2 L, 1 : 1 : 1 v/v/v), the organic layer was separated and dried over magnesium sulphate. The solution was loaded over silica gel (150 g) and the impurities were eluted with chloroform. The product was eluted with chloroform/methanol (2 : 1). The solution was evaporated under vacuum, the residue stirred with ether, filtered, and dried to give the product (18.5 g). Yield 50%.

### Z-Asp-His-D-Phe-Arg-Trp-Lys-NH_2_·3CF_3_COOH (**11**)

The fully protected hexapeptide **10** (71.5 g, 58.90 mmol) was dissolved in TFA (180 mL), and the mixture was maintained at 21°C for 40 min. The product was precipitated with ether (3 L), filtered, washed with ether, and dried to provide 72 g of the crude product **11**. Yield 80%. Purity 74% by HPLC.

### Z-cyclo[Asp-His-D-Phe-Arg-Trp-Lys]-NH_2_·2CF_3_COOH (**12**)

Linear hexapeptide **11** (300 mg, 0.197 mmol) was dissolved in DMF (30 mL), then HOBt·H_2_O (51 mg, 0.3305 mmol), NMM (65 µL), and DCC (68 mg, 0.3305 mmol) were added, and the mixture was stirred at r.t. for 24 h. The precipitate of DCU was filtered and the filtrate evaporated to dryness. The residue was dissolved in THF, filtered, diluted with AcOEt, and washed with water twice. The aqueous layer was extracted with THF/EtOAc, the combined organic extracts were evaporated to dryness, triturated with ether, filtered, and dried to give 170 mg of cyclic peptide **12**. Yield 31.4%. ESI-MS: *m/z* 502.7 [M+2H]^2+^, 1004.2 [M+H]^+^. The product was 75% pure by HPLC.

### H-cyclo[Asp-His-D-Phe-Arg-Trp-Lys]-NH_2_·2CF_3_COOH (**13**)

Protected cyclic hexapeptide **12** (8.2 g, 6.66 mmol) was dissolved in dry methanol (220 mL) and Pd(OH)_2_ (0.3 g) was added. The mixture was stirred under a hydrogen atmosphere for 20 h at r.t., then 4 h at 50 °C, filtered, and evaporated to dryness under vacuum at 40 °C to produce cyclopeptide **13** (7.0 g) as bis-trifluoracetate salt. Yield 96%.

### Ac-Nle-cyclo[Asp-His-D-Phe-Arg-Trp-Lys]-NH_2_ (**2**)

The salt **13** (7.0 g, 6.38 mmol), *N*-acetylnorleucine (1.11 g, 7 mmol), NMM (0.8 mL, 7 mmol), and HONb (1.9 g, 10.5 mmol) were dissolved in DMF (120 mL), and DCC (1.44 g, 7 mmol) was added. The mixture was stirred for 22 h at r.t., the precipitate of DCU was filtered off, and washed with cold DMF. The filtrate was evaporated to dryness under vacuum, and the residue stirred with ether, filtered, and air-dried to give 10 g of the crude material. This substance was dissolved in isopropanol/THF (1 L, 1 : 1 v/v), and washed with water (500 mL). The aqueous layer was filtered, and extracted with butanol (2 × 350 mL). The organic layer was evaporated to dryness, the residue stirred with ether, filtered, and dried under vacuum to obtain 5.2 g of melanotan II (**2**) 90+% pure by HPLC. Yield 80%. ESI-MS: *m/z* 513.1 [M+2H]^2+^, 1025.2 [M+H]^+^.
